# Integrating Large Language Models into Qualitative Methods in Health Services Research: A Proof-of-Concept Study

**DOI:** 10.21203/rs.3.rs-9726844/v1

**Published:** 2026-05-28

**Authors:** Lia Chin-Purcell, Elena Rosenberg-Carlson, Hélène Chokron Garneau, Marzan Hamid, Mark McGovern

**Affiliations:** Department of Psychiatry and Behavioral Sciences, Stanford University School of Medicine, Palo Alto, California; Department of Psychiatry and Behavioral Sciences, Stanford University School of Medicine, Palo Alto, California; Department of Psychiatry and Behavioral Sciences, Stanford University School of Medicine, Palo Alto, California; Department of Psychiatry and Behavioral Sciences, Stanford University School of Medicine, Palo Alto, California; Department of Psychiatry and Behavioral Sciences and Department of Medicine, Stanford University School of Medicine, Palo Alto, California

**Keywords:** Large language models, natural language processing, qualitative analysis, deductive coding, qualitative methods, health services research, implementation science, substance use disorder, public health, rigor

## Abstract

**Background:**

Qualitative methods are widely used in health services research to derive context-specific insights and depth of understanding. Manual coding, a foundational technique in rigorous qualitative analysis, is highly resource and time-intensive and difficult to scale. This is a particular challenge in health services research, where repeated rounds of interviews are common and rapid turnaround is often required. Natural Language Processing (NLP), specifically Large Language Models (LLMs), have shown potential to enhance efficiency in qualitative analysis. However, there is limited research providing guidance on how to integrate LLMs while maintaining rigor and trustworthiness. In this proof-of-concept study, we propose, apply, and evaluate an NLP-assisted coding method in a health services research setting.

**Methods:**

We analyzed 22 interviews among public health officials, law enforcement, community organizers, and medical professionals at one California county to examine existing substance use service gaps and needs. A primarily deductive codebook was iteratively refined until two coders achieved an inter-coder reliability (ICR) > 0.95 and was applied to the transcripts using ATLAS.ti. We developed an NLP-assisted method that uses a semantic shift algorithm to segment transcripts which are then passed to GPT-4 for code assignment and explanation using the codebook and coding guidelines developed during the manual process. We evaluated the method with a quantitative assessment of agreement between human and NLP-assigned codes, a qualitative and quantitative soundness assessment by two reviewers, and a comparative efficiency analysis.

**Results:**

The NLP-assisted method had moderate agreement with human coding (modified pooled Cohen’s Kappa = 0.66), and 71.8% of codes were rated as sound by reviewers. Sound codes were more often observed for high-certainty and straightforward codes, and when text chunks were semantically well defined. The NLP-assisted method had more difficulty with non-linear conversation and entity-dependent codes. Coding time was reduced significantly from ~40 hours for the traditional method to ~1 hour for the NLP-assisted method.

**Conclusions:**

These findings suggest that LLMs can be effectively incorporated into qualitative processes while maintaining rigor if humans are embedded into the process. By maintaining a human-in-the-loop workflow, our methodology allows for researchers to maintain familiarity with the data, define the research question(s) and codebook, and determine if there are results that are not sound. By incorporating LLMs into the coding stage of the process, key limitations of traditional qualitative methods in health services research can be addressed, such as scalability, and resource and time limitations.

## BACKGROUND

Qualitative methods are widely used in health services research. These approaches provide key insights about human experiences and contextual factors not otherwise or easily obtained with quantitative methods. In rigorous qualitative methods, coding is a foundational technique. It consists of in-depth data review and the assignment of labels to segments of text to categorize and summarize information relevant to the research question [1]. Codes act as a critical, initial connection between qualitative data and their explanation of meaning [1]. Manual coding is highly resource and time-intensive, often requiring several days to even months to complete, depending on the specific methods used and the volume and complexity of the data. Additionally, this process requires researchers skilled in the qualitative analysis process, both to lead analysis and to apply codes.

This poses particular challenges in health services research because rapid turnaround is often necessary when implementing new programs or treatment to address contextual factors or adapt interventions [2]. Additionally, qualitative methods may be used in health services research where there are rapid changes that existing quantitative datasets do not capture, and time-sensitive information is needed [3]. This is particularly important in implementation science, where insights from key informant interviews often need to be provided to participating organizations quickly to support intervention implementation efforts. Repeated rounds of interviews are common for healthcare organizations, often used to assess needs or barriers to service delivery throughout a program or study. This requires consistent, efficient methods to garner accurate and actionable information from each round of data collection [4]. Building sufficient capacity of skilled qualitative researchers creates an additional challenge, particularly for smaller and resource-limited organizations.

Researchers have been exploring the use of Natural Language Processing (NLP), computational techniques for analyzing human language, to expedite and enhance qualitative analyses [5–12]. NLP has been shown to be a potentially useful tool to analyze text data and enhance efficiency at various stages of the qualitative analysis process. While earlier NLP techniques showed promise, supervised approaches require large amounts of human-annotated data, and unsupervised approaches rarely captured the specific categories researchers intend to target [9,10,13–19]. The recent advancements of Large Language Models (LLMs), like Generative Pre-trained Transformer (GPT), Claude, and Gemini, offer increased possibilities for deriving robust insights from qualitative data quickly. Because they have a high capacity for reasoning and are flexible to various applications, they show particular promise where other NLP methods may fall short [6,20,21].

Recently, there has been an emergence of the use of LLMs to extract insights from qualitative data, and to label data given a codebook to ease the burden of time and cost [5–8,12,21,22]. Researchers have explored how LLMs can be used to generate preliminary emerging themes from qualitative data to inform an inductive coding approach, and how those themes compare to ones generated by humans [5,11,23–26]. Researchers have also explored utilizing LLMs with a deductive approach to assign labels to qualitative data based on a predefined codebook, both with pre-defined excerpts of text [27,28], and using LLMs to identify the sequence of text to which the code applies [5,7,8,29]. Aside from assigning codes, researchers have also suggested that LLMs may be used to identify ambiguous text and resolve disagreements between coders [11,26], or as research assistants [30].

Despite this growing body of literature, few studies have explored applications of LLMs in qualitative health services research, a context that presents unique considerations for the application of LLMs. First, deductive coding approaches are common because research questions are typically tied to established theoretical frameworks or aimed at answering specific questions relevant to service delivery. Second, health services interviews are often greater than 30 minutes in length, producing transcripts too long to pass to an LLM in a single input. Taken together, these elements necessitate a workflow that allows text to be processed in smaller, meaningful segments and then consistently applies a deductively derived codebook.

Existing literature does not adequately address these needs. The majority of aforementioned studies using LLMs for qualitative analysis did not use interview data at all, instead applying LLMs to structured text such as social media posts, forum comments, historical passages, or survey responses that require no segmentation [5–8,11]. Social media posts, journalistic text, and other public-facing content appear particularly well represented in LLM training data, meaning LLMs may perform better on these data compared to real-world interview transcripts. Among studies that did use interview transcripts, the text was either passed to the LLM as a whole or divided based on context window constraints [12,22,24,26,29]. Some studies were conceptual and did not apply LLMs to empirical data [22,25,30]. Also, few studies have evaluated an LLM-assisted coding workflow and output using qualitative rigor criteria [31,32]. The primary goal of this study is to help address these gaps by proposing and evaluating a new method for the application of LLMs in qualitative health services research.

The use of LLMs in qualitative research also raises important questions about rigor and trustworthiness. In qualitative research, the human researcher is traditionally considered the analytic instrument. The ontological and epistemological principles of traditional qualitative research as well as several techniques to establish rigor and trustworthiness in qualitative research, such as prolonged engagement with the data and memoing, are predicated on a human analytic process [33]. How LLMs can be used in qualitative analysis is very early in its research developmental stage, and there is no standard of practice for how LLMs are best used in the analysis process. Because of this, guidance and methodology are needed to determine how best to integrate LLMs while maintaining rigor and trustworthiness of results. Additionally, methodologies are needed in which researchers are engaged with the text and can review results for credibility. Therefore, a secondary goal of this study is to demonstrate how qualitative rigor and trustworthiness can be preserved when LLMs are integrated into the analytic process.

## METHODS

### Dataset

The dataset comprises 22 interviews among public health officials, law enforcement, community organizers, and medical professionals in one county in the State of California. The interviews are semi-structured and were conducted with the goal of gaining insight into the existing substance use disorder (SUD) service network and gaps and needs within the county. Participants were recruited using a snowball sampling method where a small sample of initial participants was identified, and additional participants were identified by asking who else might have information on SUD services in the county. The interviews were conducted via videoconference over the course of 10 weeks in 2023 and were recorded with the consent of the interviewees. The interviews were transcribed by a professional transcription service which also removed the names of the interviewees and assigned participant IDs. The recordings and transcriptions were securely stored on the Stanford Medicine Box. The interviews are on average 8,301.6 tokens, 12.8 transcribed pages (12pt font single spaced), and about an hour in length.

### Traditional Coding Method

Our traditional coding method involved 2 human coders, 1 of whom is the first author (LCP), and 1 arbiter, an experienced qualitative researcher who is the second author of this paper (ERC). A primarily deductive coding method was used, with an initial codebook and coding guidelines developed by the arbiter based on the project objectives and interview guide and then refined in collaboration with the 2 coders via an iterative process. The interview guide was developed for this study and is provided as Additional File 1 – Interview Guide. The codebook and accompanying coding guidelines were independently applied to selected interview transcripts using ATLAS.ti (version 25.0.2; ATLAS.ti Scientific Software Development GmbH) [34]. The codebook and coding guidelines were revised through multiple rounds of discussion and reliability testing until high intercoder agreement was achieved (Cohen’s kappa > 0.95) [35]. After intercoder reliability was established, the 2 coders each independently coded 11 transcripts using the finalized codebook and coding guidelines.

### NLP Methods Tested

Several NLP methods were identified and evaluated for viability prior to developing the final workflow. Details of this initial evaluation are provided in Additional File 2 – NLP Methods Tested. Ultimately, a semantic shift segmentation algorithm was developed for segmenting long interview transcripts into meaningful units, as it identifies topic changes using embedding-based similarity rather than relying on paragraph boundaries or fixed character windows [36].

### Finalized NLP-Assisted Method Workflow

Our approach outlined here incorporates the use of LLMs in a way that is iterative and collaborative with researchers. We aim to preserve human engagement in and leadership of the coding process while utilizing LLMs to enhance efficiency and make the process more scalable for larger amounts of data. Also, we consider the limitations of LLMs, such as hallucinations, context window issues, and non-specialization, and provide a workflow where these limitations are mitigated. The workflow is primarily model agnostic, with the hope that it continues to be useful as others consider how LLMs can be incorporated into their coding process.

**Figure 1.**
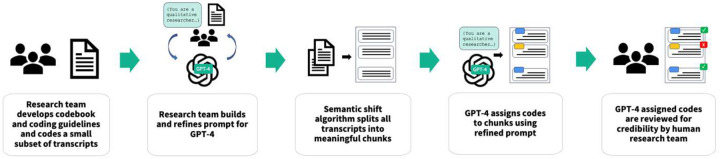
NLP-Assisted Method Workflow

We demonstrate our approach in a proof-of-concept format where we show its application to our dataset of 22 interview transcripts from one qualitative study. We present a developed “NLP-assisted” method which incorporates LLMs into the workflow as well as other NLP methods including SentenceTransformer embeddings. The overall structure of the proposed method is to split the transcripts up into meaningful “chunks” using a semantic shift algorithm, which are then passed to GPT-4 for coding, along with the codebook and coding guidelines (see Figure 1). We explain the details of the proposed method below.

### Step 1. Research team develops codebook and coding guidelines and codes a small subset of transcripts

The first step of our method involved a human research team developing a codebook and coding guidelines to code a subset of randomly selected transcripts. In this step, the human research team first generated a codebook and coding guidelines based on the interview guide and research question. Then, they iterated on the codebook and coding guidelines until consensus is reached amongst the coders.

By coding a subset of transcripts, the human research team preserved familiarity and engagement with the data, which are key aspects of qualitative research [1]. They were also better equipped to review the codes applied by the model for credibility.

### Step 2. Research team builds and refines prompt for GPT-4

In the next step, the codebook and coding guidelines were used to develop a prompt for the LLM. When designing the prompt, researchers have found that crafting a useful prompt to the LLM is key to obtaining good results, specifically assigning the model a role and specifying what the output should look like [26]. We began with human-identified text excerpts rather than automated segmented text, allowing us to iterate on prompt wording without the added complexity of chunking issues.

**Figure 2.**
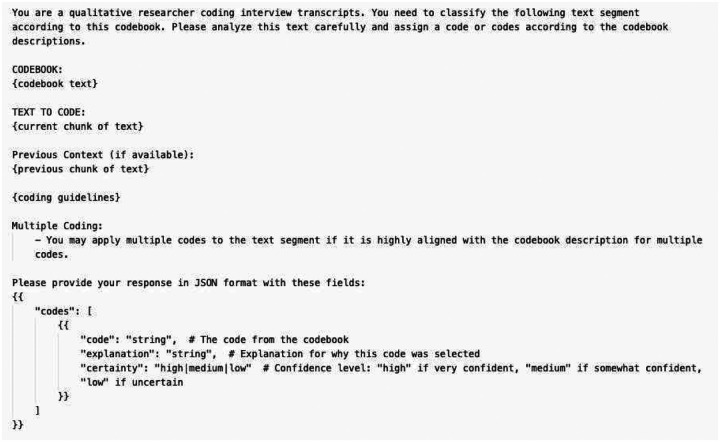
Prompt Structure Overview

In our case, our prompt was structured by first stating the task for the LLM: *“You are a qualitative researcher coding interview transcripts. You need to classify the following text segment.”* Next, we proceeded with the codebook, the current chunk of text to code, the previous chunk of text (if available) for context, and the coding guidelines developed in the previous step (see Figure 2). The LLM is asked to provide its response in structured, machine-readable format (JSON) format, and for each code assigned we asked for a certainty indicator (high, medium, low) and an explanation for why the code was assigned.

The prompt was applied to each chunk of text identified by the human coding. The results of this coding are then compared to the codes applied by the humans for the purpose of iterating on the prompt to enhance the LLM’s ability to apply codes as intended by the research team. For example, we initially observed an under-coding of parent codes compared to the manual codes applied. To address this, we included the term “(Parent Code)” in the code name for those codes, which resulted in the LLM more frequently making appropriate use of the parent codes. We repeated this process until satisfactory agreement was reached between the LLM coding and the human coding, and the human research team had sufficient confidence in the LLM’s ability to appropriately apply the codes.

### Prompt Iteration with Semantic Shift Chunks

The LLM prompt was developed iteratively across 5 transcripts (22% of the dataset), with refinements including reducing emphasis on interviewer questions, improving guidance for context-specific codes, and determining the optimal number of codes to request per chunk. Full details of the prompt iteration process are provided in Additional File 3 – Prompt Iteration, and the finalized codebook and coding guidelines in the prompt are provided in Additional File 4 – Codebook.

### Step 3. Semantic shift algorithm splits all transcripts into meaningful chunks

Once the prompt was finalized, the semantic shift algorithm was applied to all transcripts to split them into meaningful chunks. In our implementation, we used the SentenceTransformer model all-mpnet-base-v2 to generate vector embeddings of each paragraph [37]. Our transcripts were split into paragraphs by our transcription service that were on average 3.5 sentences in length. We added two question-detection rules to ensure that interviewer questions and their responses were kept together in the same chunk, and that new questions initiated a new chunk.

We made 5 additional rounds of iteration on the prompt at this stage, before making the determination that the method displayed sufficient ability to code the chunks as intended by the research team and proceeding with the whole corpus of transcripts.

### Step 4. GPT-4 assigns codes to chunks using refined prompt

Once transcripts were segmented, the refined prompt was applied to each chunk to assign codes. For transcripts that contain Protected Health Information (PHI) or other sensitive data, this step should be completed using a secure or local instance of an LLM to preserve privacy and security. An API was also used within a Python script to automate calls to the LLM across all chunks, rather than manually submitting them, to create a streamlined and efficient process.

**Figure 3.**
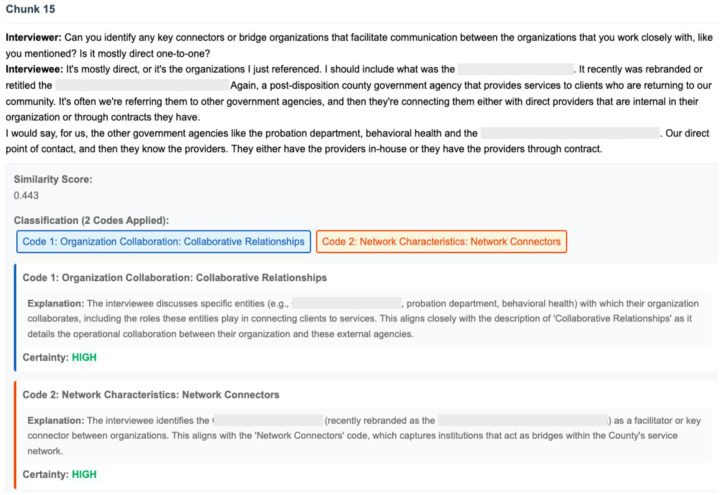
Example coded chunk

In our case, we used a secure instance of GPT-4 hosted by an academic medical center for all our LLM calls. This academic medical center has deployed a secure, private instance of GPT-4 referred to as Secure GPT. Unlike public-facing LLMs like ChatGPT, Secure GPT is configured to handle Protected Health Information (PHI) safely and securely using a private infrastructure and closed network access [38]. The result was each chunk receiving 2–3 codes on average, along with an explanation and certainty indicator for each code (see Figure 3). Table 1 shows the distribution of chunks by number of codes assigned across all transcripts. Because the output was stored in JSON, coded chunks could then be organized by code or parent code for downstream analysis.

### Step 5. GPT-4 assigned codes are reviewed for credibility by human research team

As a final step, the research team reviews the final codes applied by the LLM for credibility. The goal of this step is similar to an arbiter’s review process to ensure that codes are consistently applied appropriately based on alignment between the chosen code(s) for a given chunk and the coding frame and guidelines. During this step, the research team may correct certain codes and note patterns or concerns. This step is crucial because the LLM may occasionally hallucinate, and the human research team would be equipped to audit the codes applied due to their experience with applying the codebook without the use of an LLM. Researchers doing this step benefit from having coded a subset of transcripts in Step 1, as that familiarity with the codebook helps them to assess the model's output. In our case, the research team reviewed a subset of transcripts LLM-assigned codes for evaluation purposes detailed below.

### Evaluation of the NLP-Assisted Method

To critically evaluate our proposed method, we conducted both a quantitative assessment of agreement between human coders and the NLP-assisted method, and a qualitative assessment of the soundness of the codes assigned by the NLP-assisted method. We conducted both assessments to have a direct comparison against human coders while also recognizing multiple sound perspectives may exist regarding which code(s) to apply, consistent with the constructivist lens underpinning most traditional qualitative research. An efficiency analysis was also conducted to compare time required to apply codes using the NLP-assisted method versus the manual method.

### Quantitative Evaluation of Coding Agreement

To assess quantitative agreement between the human coders and the NLP-assisted method, we computed a modified version of Cohen's kappa. The semantic shift algorithm segments the entire transcript and GPT-4 assigns a code to every chunk. In contrast, human coders were instructed to code only relevant content, exercising judgment about whether to include interviewer questions based on context. As a result, the NLP-assisted method inevitably produced sections with LLM-assigned codes that received no corresponding human code. Therefore, we computed a modified kappa which measures recall-oriented agreement, that is, how well the LLM captures what humans coded, rather than general inter-coder reliability. We also report the total proportion of rows and characters that were LLM-only coded across transcripts. To compute the modified kappa, we identified all start and end character positions from both LLM and human codes. Each row in the dataset corresponded to a section with start and end positions, and the corresponding semicolon-separated LLM codes and human codes. Rows where there were only LLM codes were removed. Post-processing filters were applied to remove duplicate rows and very short sections.

Because GPT-4 code assignment could contain multiple codes per section, GPT-4 codes were split into their individual codes and directly compared to the human code. If the human code matched any of the GPT-assigned codes, the section was treated as an agreement. If the human code did not match any LLM code, the section was treated as a disagreement. 2 variants of the modified Cohen's kappa were computed for each transcript, as well as a pooled Cohen's kappa. The first variant of Cohen’s kappa retained all codes, and the second had the “Miscellaneous” code removed, a codebook category used to capture content deemed relevant but not fitting any other code. The pooled Cohen’s kappa is computed by aggregating all sections across all transcripts and computing the metrics for all chunks together. Evaluation was performed on all transcripts. Credibility of the NLP-assisted method findings was further addressed through the soundness assessment reported below.

### Qualitative and Quantitative Evaluation of Coding Soundness

In addition to the quantitative assessment of agreement, a quantitative and qualitative assessment of reasonableness or soundness of the GPT-4 codes assigned to segments was conducted. This assessment accounts for the possibility that the codes applied by GPT-4 may be different than the ones applied by humans, while still being reasonable given the codebook, coding guidelines, and provided explanation.

A subset of 5 transcripts was selected to ensure representation of different interviewee roles across the county SUD service network. The qualitative assessment was completed by 2 members of the research team who are also the first and second author of the paper. Both reviewers assessed the first 3 codes applied to each chunk of the 5 transcripts and evaluated the code for 1) soundness with respect to the codebook, 2) soundness with respect to the coding guidelines, and 3) soundness of the provided explanation. A binary rating of “sound” or “not sound” was applied to each code based on these requirements, as well as a short 1–2 sentence justification. After both reviewers independently rated each code applied by the NLP-assisted method, we reviewed our ratings together to come to a consensus and discussed patterns observed. We then calculated overall soundness as the percentage of sound codes among all reviewed codes and computed this metric separately for high certainty and medium certainty codes.

### Comparative Efficiency Analysis

We compared the amount of time taken for the NLP-assisted method to complete its coding of the 22 interviews compared to the amount of time it took for a human research team to code the interviews. We measured the time spent applying the codebook to the interviews rather than the time spent developing the codebook and coding guidelines because these outputs are also used in the NLP-assisted approach. Since coding is typically the most time-intensive step in qualitative analysis, we focused on this phase to have the greatest potential for increase of efficiency while preserving key aspects of the traditional coding process.

A multi-threaded approach with back-off was implemented to make API calls to Secure GPT to increase efficiency by handling multiple requests while also avoiding rate-limiting errors. Total processing time for the manual method and the NLP-assisted method were recorded and compared.

## RESULTS

### Quantitative Evaluation of Coding Agreement

Agreement between human coders and the NLP-assisted method was assessed using 2 variants of the modified Cohen’s kappa. The modified kappa reflects how well the NLP-assisted method captured what humans coded, rather than a conventional inter-coder reliability metric. Generally, recall-oriented agreement between human coders and the NLP-assisted method was moderate to substantial. With all codes included, the modified Cohen’s kappa ranged from 0.45 to 0.85 (mean = 0.63; median = 0.63, IQR = 0.57–0.69) with a pooled agreement across transcripts of κ = 0.63. When the miscellaneous code was excluded, agreement improved slightly with kappa values ranging from 0.54 to 0.85 (mean = 0.67; median = 0.64, IQR = 0.61–0.70) with a pooled agreement of κ = 0.66. Across all transcripts, LLM-only coded rows represented 46.8% of total rows and 24.2% of total characters, reflecting the method's design of coding all content.

### Qualitative and Quantitative Evaluation of Coding Soundness

Across the 5 transcripts reviewed, 71.8% (295 / 411) of the codes applied by the NLP-assisted method were rated as sound based on the codebook, coding guidelines, and provided explanation. Codes that were rated sound accurately reflected the definitions in the codebook and appropriately cued into the entity being discussed in a given chunk. The explanation provided by GPT-4 was often grounded in the text and aligned with what the reviewers would have used to assign a code. Additionally, explanations often acknowledged interpretation or implications for codes assigned that were less explicit, which aided in review. If a code was high certainty, it was more likely to be sound (82%, 244/296); however, there were medium certainty codes that were also rated as sound (45%, 51/113), and not all high certainty codes were rated as sound. The one low certainty code reviewed was not sound.

Some codes assigned that were more consistently sound were more straightforward and discrete codes (formatted as Parent Code: Code) such as “Organization Characteristics: Organization Resources” (90.91%, 10/11) and “Organization Characteristics: Populations of Focus” (87.50%, 21/24). On the other hand, codes with lower soundness percentages often were more complex and relational, for example “Organization Collaboration: Collaboration Facilitators” (54.5%, 6/11) and “Network Characteristics: Communication Patterns” (55.6%, 5/9). Generally, when the chunks of text were semantically well-defined, included a question from the interview guide that mapped directly onto a code, and the interviewee response was directly related to that question, the codes applied by the NLP-assisted method were found to be sound.

One common reason codes were rated as not sound was because another code unequivocally was more appropriate for the given chunk. For example, a chunk included the interviewer asking about funding a resource allocation. The response included discussion of budgets and allocation at the county and discussed opioid settlement funds used for opioid and substance use-related services. GPT-4 applied both the “Organization Characteristics: Organization Resources” code and the “Organization Characteristics: Opioid Use Services” code, and in this case, we reasoned that “Organization Characteristics: Organization Resources” was unequivocally more appropriate. Therefore, the additional application of “Organization Characteristics: Opioid Use Services” was not sound. Relatedly, we found that generally GPT-4 applied more codes than humans overall, and the “Opioid Use Services” code was particularly over-coded, as well as “Community Needs.”

Some of the coding assignments by GPT-4 that were not sound required an understanding of the specific entity being discussed. For example, there were instances in which a vague pronoun reference such as “we” was used to refer to the interviewee’s organization, and GPT-4 assigned a code as if they were referring to the service network broadly.

Another reason codes were rated as not sound was that given the explanation, GPT-4 seemed to be referencing the previous chunk too heavily and assigning a code that would be appropriate for the previous chunk, rather than using the previous chunk only for context. We also observed that sometimes, due to irregular conversation, there were chunks that were not semantically well-defined such as a back-and-forth end of conversation, or only including the interviewer question. Here, “Miscellaneous” might be the most appropriate code, however GPT-4 might assign a code based on the previous chunk or assign a code based on the question asked. Overall, we observed that the “Miscellaneous” code was under-coded.

Occasionally, the model hallucinated a code or slightly changed the code name. For example, “Network Characteristics: Network Challenges” became “Network Characteristics: Network Gaps,” “Network Characteristics: Network Resources” was made up (our codebook included “Organization Characteristics: Organization Resources”, but not “Network Characteristics: Network Resources”), “Organization Collaboration” and “Organization Characteristics” lost their parent code indicators, and sometimes the model mixed up parent and child code relations. Once, the model did not assign a code. There were also a few more benign errors, like making populations in “Organization Characteristics: Populations of Focus” not plural or removing the parent code designation (“Organization Characteristics (Parent Code)” becoming “Organization Characteristics”). For these more obvious errors, we conducted post-processing to align the code names.

### Comparative Efficiency Analysis

The total time required for the NLP-assisted method was 57 minutes and 40 seconds. The minimum time it took to code a transcript was 1 minute and 34 seconds, and the maximum time was 4 minutes and 15 seconds. In contrast, human coders took 39 hours and 15 minutes to code the same interviews. On average, the NLP-assisted method took 2 minutes and 30 seconds per transcript, compared with 107 minutes and 2 seconds per transcript for humans.

## DISCUSSION

Overall, our findings demonstrated moderate-to-substantial recall-oriented agreement between human coding and the NLP-assisted method (κ = 0.66), and that the NLP-assisted method assigned codes that were deemed “sound” a high portion of the time (71.8%). However, we also observed some limitations of the NLP-assisted coding method, namely the semantic shift algorithm struggling to segment the transcript into meaningful segments when conversational flow was very non-linear and unstructured, and the GPT-4 tending to over-code some codes and occasionally hallucinating (providing a code or explanation that was not logically related to the context provided).

### Strengths of the NLP-assisted Method

#### Efficiency and Consistency

The NLP-assisted approach demonstrated a significant reduction in coding time compared to the human coders, indicating a gain in efficiency. Methods utilizing LLMs can code many transcripts quickly, which is especially helpful for projects with large amounts of data. Scaling up to even hundreds of transcripts might be feasible for a small research team when it otherwise would not be possible due to time and capacity constraints. In addition to efficiency gains, the NLP-assisted approach applied codes relatively consistently across all transcripts, reducing the variability that can arise between human coders or over time.

It is important to note that there are time and expertise requirements to implement the NLP-assisted approach. Once an NLP-assisted workflow has been established, it would be relatively easy to adapt it to new research questions and new codebooks, enhancing flexibility and efficiency in the process. While this workflow requires an initial investment in infrastructure setup, subsequent rounds of interviews can be analyzed more efficiently, making it well-suited to health services organizations that conduct repeated interviews (see Table 2).

#### Sound Coding

Based on our quantitative soundness assessment, we found that there was a relatively high proportion, 71.8% (295/411), of codes that were applied reasonably given the codebook and coding guidelines. These findings are broadly consistent with other studies utilizing LLMs for deductive coding, which have reported fair to substantial kappa values [5,6,8] and accuracy rates ranging from approximately 66–90% [5,7], though performance varies by data type and task complexity. This shows promise for GPT-4’s ability to appropriately apply codes when the prompt is adapted for a particular coding task. While oversight of coding is still required with an NLP-assisted method, the output provides a structured set of codes that researchers can efficiently review and use for further analysis.

### Challenges of the NLP-assisted Method

#### Semantic Shift Algorithm Limitations

The semantic shift algorithm struggled to segment meaningfully when the text was less structured. We found that including a series of rules for detecting questions and answers, clarifications, short responses, and using a more robust Sentence Transformer model for embeddings improved performance. However, these rules are hard coded into the algorithm and ultimately are subject to the nuances of natural language and the non-linear nature of human conversation.

When the algorithm struggled to segment meaningfully and provide appropriate context, GPT-4 tended to struggle to assign appropriate code(s). Many incorrect coding assignments resulted from not cueing into the appropriate entity. In some cases, to make a sound code application it was essential to understand context that may have been embedded throughout the interview. In other cases, the appropriate context was included, but understanding the entity being discussed was difficult or interpretive, which humans also struggled with. This suggests that algorithms used to segment transcripts may work better for structured interviews. If interviews are more free-form, obtaining segments during transcription may be more efficient and yield better results.

#### Over-coding

Based on our qualitative review of the GPT-4 codes, we found that GPT-4 tended to apply too many codes to the chunks as reflected in the 46.8% of rows that were only coded by the LLM. Despite instructions in the prompt to only apply secondary codes if they were highly aligned with the codebook, GPT-4 sometimes assigned lower certainty second or third codes to chunks where reviewers determined the first code was unequivocally more appropriate. This tendency may show an over confidence in GPT-4’s responses, which has been demonstrated for LLMs in the literature [26].

The over-coding of the “Organization Characteristics: Opioid Use Services” and “Community Needs” codes may be due to the specificity of the topic; the goal of the project was to explore the landscape of substance use services, so words related to substance and opioid use are common throughout the interviews. GPT-4 seemed to code the “Organization Characteristics: Opioid Use Services” code when substance use related words appeared, but it was aimed at capturing a specific part of the interviews where the interviewees discuss the services they provide. Similarly, the “Community Needs” code was aimed at capturing information about communities that face barriers to accessing services, which was a specific question in the interview guide. GPT-4 seemed to apply this code whenever a population or community was mentioned (where “Organization Characteristics: Populations of Focus” may have been more appropriate), or an area where there may be a gap in services (where “Network Characteristics: Network Challenges” may have been more appropriate). This reliance on keywords rather than contextual meaning is consistent with the finding that LLMs perform better on context-independent than context-dependent coding tasks [8]. This is also an example of a potential shortcoming of using a general model such as an out of the box large language model like GPT-4 for a relatively specific area of focus. One way that this could be addressed is by fine-tuning the model with material from the specific context before assigning codes.

#### The Role of LLMs in Qualitative Research

Many qualitative researchers argue that the interpretive, meaning-making process of qualitative research is and should remain a distinctly and exclusively human practice [39]. We agree that humans have an essential role to play in qualitative analysis, and do not propose the uncritical replacement of traditionally human analytic activities with LLMs. We likewise believe that traditional, reflexive “Big Q” Qualitative analysis approaches are uniquely valuable in their ability to produce in-depth, nuanced understanding of people and social processes. We do not suggest that our methodology should be considered as equivalent, nor that an automated process should be applied uncritically in circumstances where this is the goal. However, in fields such as health services research where obtaining results quickly is a high priority, NLP-assisted approaches may be useful. Given the accelerating use of LLMs in qualitative research, developing methodologies that preserve trustworthiness is both timely and necessary.

### Limitations

While this proof-of-concept allowed us to understand what some of the strengths and challenges are to using an NLP-assisted coding method, it should be noted that the dataset used is comprised of a relatively small set (22) of interviews, in a relatively specific subject area. The unique dataset may limit the generalizability of the proposed method’s results to other qualitative research projects.

Additionally, we report a modified Cohen's kappa to provide a quantitative assessment of the agreement between human coders and the NLP-assisted coding approach. Because this metric is modified, it may not be directly comparable to standard measures of agreement, for example between two human coders. We modified Cohen's kappa to account for the differences in coding application: the NLP-assisted method codes all segments of the transcript and can apply multiple codes per section, while human coders selectively code text and are more likely to double code overlapping sections rather than assign multiple codes to the same section. Our modified version of Cohen's kappa captures agreement and disagreement between human and the NLP-assisted coding, but also avoids large penalties for expected differences, such as the NLP-assisted method coding more text than the humans, or humans not applying all codes to a particular part of text that the NLP-assisted method has chosen.

## CONCLUSIONS

Our results suggest that LLMs may be able to be effectively utilized for qualitative coding to increase efficiency of qualitative analysis processes while maintaining trustworthiness. The importance of maintaining human oversight and engagement in LLM-assisted coding has been emphasized in the broader literature, with researchers recommending iterative prompt refinement, human oversight, and review of outputs for credibility [6,26,31,32]. Low-resourced settings where there may not be enough analysts or time to code and analyze qualitative data could benefit particularly from this workflow, whereby using this methodology, they could ask research questions that they wouldn’t have otherwise been able to pursue. Additionally, in health services research settings, where repeated rounds of interviews are common, a rigorous workflow that can be efficiently repeated is particularly valuable.

Future research should test the method with other types of data and larger pools of data to assess applicability for other settings and research questions. It would also be useful to conduct usability testing of the method itself and how researchers find its ease of use and how it can be improved. Also, there may be several ways that NLP and LLMs specifically can arrive at themes and insights using more of an inductive approach, or may deviate from the traditional qualitative process more, for example not applying codes to transcripts per se but rather retrieving relevant information or summarization. For example, Retrieval Augmented Generation (RAG), which grounds LLM outputs in retrieved passages from the dataset, has been explored to assist with the analysis of interview transcripts. This method moves away from code application toward retrieving relevant information from the data and generating summaries [40].

New LLMs and associated methodologies are being developed quickly, and best practices and strategies change frequently. As new models are released, new methodologies may work better or worse depending on the strengths of the model. Currently, using LLMs within Computer-Assisted Qualitative Data Analysis Software (CAQDAS) such as ATLAS.ti often lacks transparency and can be difficult to modify or tune to researchers’ needs. However, these processes could be improved to enable smoother integration of LLMs into traditional coding workflows. As the field continues to develop methodology using LLMs to assist with qualitative research, this proof-of-concept offers a human-centered workflow grounded in qualitative rigor. This methodology has the potential to expand research possibilities for qualitative health services researchers under time and resource constraints.

## Supplementary Material

Supplementary Files

This is a list of supplementary files associated with this preprint. Click to download.

- AdditionalFile1InterviewGuide.docx
- AdditionalFile2NLPMethodsTested.docx
- AdditionalFile3PromptIteration.docx
- AdditionalFile4Codebook.docx

## Figures and Tables

**Table 1.** Distribution of Chunks by Number of Codes

Number of Codes	Chunks, n (%)
1	110 (13.8%)
2	399 (49.9%)
3	218 (27.3%)
4	59 (7.4%)
5	10 (1.3%)
6	3 (0.4%)

**Table 2.** Setup Activities vs. Repeated Activities

One-time setup	Per-round of qualitative interviews
Develop codebook and coding guidelines	Run automated semantic shift
Humans code small subset of transcripts	Run automated coding
Develop prompts for LLMs through iteration	Humans review automated coding
Adapt semantic shift method for transcripts	

## Data Availability

Raw interview data are not publicly available to protect the privacy of participants and in accordance with IRB restrictions. The code used to implement the NLP-assisted method described in this article is available in the following repository [41]: Project name: NLP-Assisted Qualitative Coding Method Project home page: https://github.com/liasop/nlp_assisted_method Archived version: https://doi.org/10.5281/zenodo.19896562 Operating system(s): Platform independent Programming language: Python License: MIT Any restrictions to use by non-academics: None

## LIST OF ABBREVIATIONS

NLP: Natural Language Processing
LLM: Large Language Model
GPT: Generative Pre-trained Transformer
SUD: Substance Use Disorder
IRB: Institutional Review Board
JSON: JavaScript Object Notation
PHI: Protected Health Information
API: Application Programming Interface
IQR: Interquartile Range
RAG: Retrieval Augmented Generation
CAQDAS: Computer-Assisted Qualitative Data Analysis Software
